# Study on mechanical behavior and damage process of concrete with initial damage under eccentric load

**DOI:** 10.1038/s41598-021-95964-x

**Published:** 2021-08-12

**Authors:** Ge Lina, Yi Fu, Zhou Junxia, Du Changbo

**Affiliations:** 1 College of Architecture and Transportation, Liaoning Technical University, No. 88 Yulong Road, Xihe District, Fuxin, 123000 Liaoning People’s Republic of China; 2 School of Civil Engineering, Liaoning Technical University, No. 88 Yulong Road, Xihe District, Fuxin, 123000 Liaoning People’s Republic of China

**Keywords:** Structural materials, Civil engineering

## Abstract

In order to analyze the influence of eccentric load on mechanical properties and damage process of concrete with initial damage, the eccentric load compression tests of concrete under different confining pressures were carried out with the help of PFC particle flow program. The results show that: the eccentric load does not change the relationship between peak stress, crack initiation stress and confining pressure of concrete under uniform load, but decrease the value of them. The peak stress increasing coefficient under uniform load is higher than that under eccentric load, and the peak stress increasing coefficient increases in a linear function with the confining pressure, and the increasing rate is approximately the same. Under uniaxial compression of eccentric load, a type I shear crack approximately parallel to the loading direction is formed, while under biaxial compression, a bending type shear crack with the lower tip of the initial crack as the inflection point is formed. The number of microcracks in concrete under uniform load and eccentric load can be divided into three stages: the calm period at the initial loading stage, the pre-peak expansion period from crack initiation point to peak point, and the rapid increase period after the peak.

## Introduction

**Figure 1:**
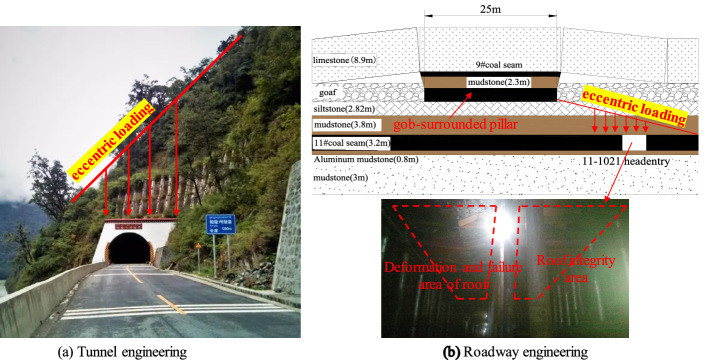
The phenomenon of eccentric loading in engineering.

In practical engineering, most concrete structures have experienced a certain load history, such as the water level change of dams, the load change of bridges, the dynamic load action of buildings such as earthquakes or explosions. The existence of load history will produce micro-cracks of different sizes in concrete, that is, the initial damage. The existence of initial damage will affect the deterioration process of concrete damage and reduce the service life of concrete^1,2^. The concrete in the tunnel or the coal and rock mass in the sinking and driving engineering are often subjected to eccentric load due to the undulation of the mountain or the excavation of the project (Fig. 1). The internal stress state, damage deterioration process and deformation field evolution law of concrete, rock and other solid materials under eccentric load are different from those under uniform load^3,4^. Therefore, the research on the mechanical behavior and damage law of concrete with initial damage under eccentric load is of great significance for mastering its macroscopic mechanical properties and guiding field engineering practice. Meanwhile, it is also helpful to establish a constitutive model that can accurately describe the complex mechanical properties of such media under actual engineering conditions.

On the mechanical properties and damage mechanism of concrete, scholars all over the world have carried out extensive research and achieved fruitful results. Yu et al.^5^ carried out full area and local area multiaxial compression tests on ordinary concrete, compared the failure modes of concrete under different loading conditions, and proposed the failure criteria of ordinary concrete under local load based on the true triaxial compression test data. Zingg et al.^6^ studied the influence of cement matrix porosity on the triaxial properties of concrete under high confining pressure, and considered that the contribution of cement matrix porosity to concrete strength decreased under high confining pressure, which enriched the research results of triaxial compression of mass ordinary concrete. Wang et al.^7^ studied the influence of water saturation and loading rate on the performance of dam concrete under biaxial compression, and considered that the ultimate strength of dry and saturated concrete increased with the increase of strain rate, while the damage pattern and ultimate strength were closely related to the magnitude of lateral pressure exerted on the specimen. The dynamic failure criterion was proposed to characterize both the effects of strain rate and water content on the ultimate strength of dam concrete under biaxial compressive stress states. Tian et al.^8^ studied the damage characteristics of concrete under uniaxial compression using X-ray computed tomography (CT), The process of concrete crack initiation, propagation, penetration and failure under uniaxial compression was given by using CT imaging technology, and the damage fracture zoning theory for quantitative analysis of concrete damage evolution was proposed by using CT. With the continuous development of computer technology, numerical simulation method has been widely used in the study of mechanical damage characteristics of materials. Ansari et al.^9^ used particle flow code (PFC) software based on discrete element method to simulate the performance of pervious concrete specimens with different aggregate size and void ratio. By comparing with the test results, the reliability of using discrete element method to simulate the mechanical properties of pervious concrete was proved. Lian et al.^10^ used PFC to simulate the mechanical behavior of porous concrete under compression and tension. The accuracy and effectiveness of the simulation were verified by comparing with the experimental results and empirical formula. Huang et al.^11^ used the particle flow code (PFC) to simulate the strength failure behavior of three non- coplanar specimens under uniaxial loading, and four typical crack merging modes were determined, namely shear, tension shear mixing and tension. Through the analysis of the force field and displacement field, the evolution mechanism of cracks around the holes of granite specimens was revealed. In the framework of PFC, Song et al.^12^ proposed a three-dimensional multi-level stress corrosion model (MSC) to reproduce the mechanical properties of brittle geo materials and concrete under multi-level cyclic compression loading. The simulation results of MSC model are in good agreement with the experimental results. The results show that the MSC model can reproduce the cyclic loading mechanical properties of concrete specimens under different maximum and minimum load levels.

To sum up, scholars from all over the world have carried out extensive research on the mechanical properties and damage process of concrete under uniform load, but the research on the damage process of concrete under eccentric load is rarely reported, and most of the existing research is based on complete concrete samples, while the research on the mechanical law of concrete with initial damage is rare. Based on this, the mechanical properties and damage laws of concrete with initial damage under uniform load and eccentric load are studied by means of particle discrete element method on the basis of parameter calibration. The research results have important guiding significance for the engineering design of concrete and the formulation of monitoring early warning indicators.

## Simulation scheme of particle flow code

In this paper, PFC2D is used to simulate the eccentric compression test of concrete. PFC simulation software is based on DEM discrete element method, and the basic mechanical properties of materials are described by the motion of each particle and the force and moment acting on each contact point. Concrete is usually composed of a group of particles bonded by parallel bonds. The strength of concrete is achieved by parallel bonding between particles, and its essence is the cementation between particles. The program can track and show the crack initiation and growth process, and can distinguish the tensile crack and shear crack, which is widely used in concrete and other solid materials^13–15^.

### Establishment of numerical model

**Figure 2:**
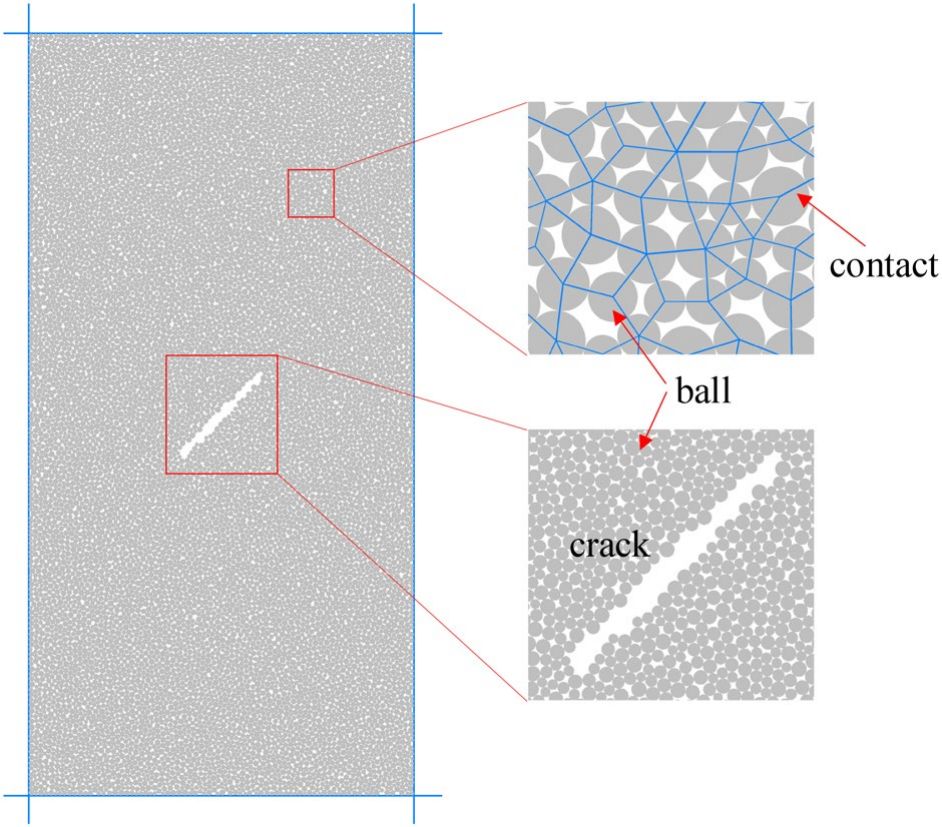
Numerical model of concrete with initial damage.

The numerical simulation model is consistent with the indoor test. The sample size is 100 mm in height, 50 mm in width, 0.25–0.415 in particle radius, and the number of particles is 12,392. The parallel bond contact model is used and the number of contacts is 23,682. In this paper, the initial fracture is established by deleting particles. The length of the initial fracture is 15 mm, the width is 0.5 mm, and the angle between the initial fracture and the horizontal direction is 45 degrees. The final numerical model is shown in Fig. 2.

### Loading mode

**Figure 3:**
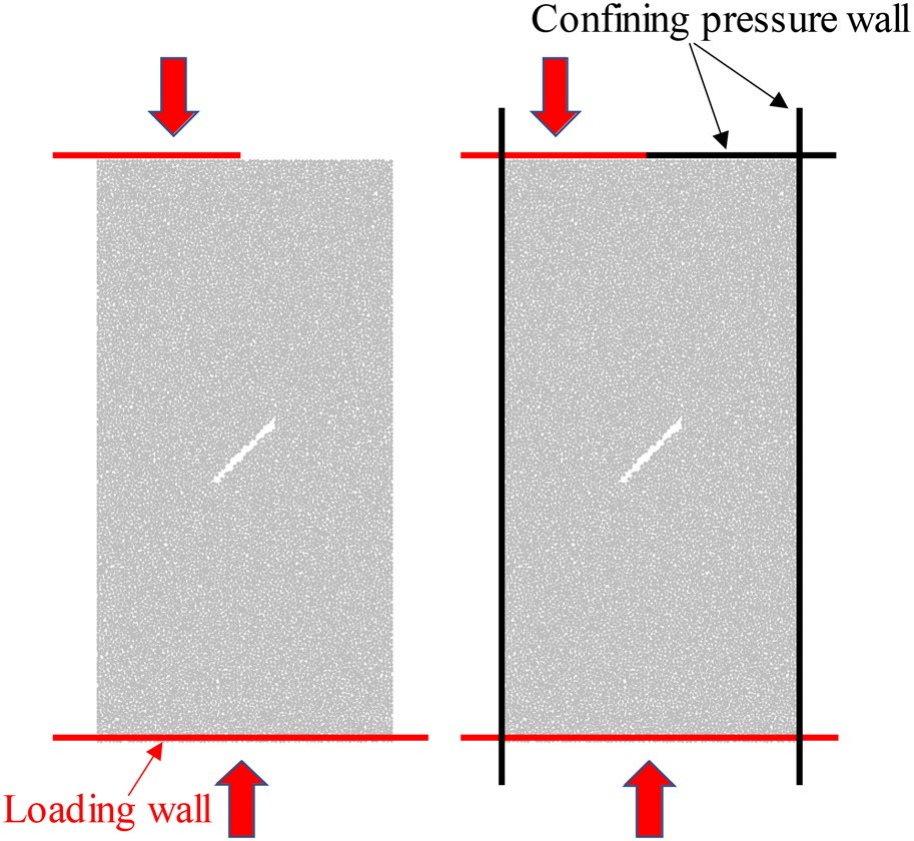
Schematic diagram of eccentric load loading.

The displacement loading method is adopted. The upper and lower walls are loading walls, and the moving speed of the wall is set to 0.05 m/s. According to the existing literature research results^16,17^, the set wall velocity can simulate the quasi-static loading in laboratory test, and the loading will stop when the post peak stress drops to 80% of the peak stress. When the uniformly distributed load is compressed, the load is applied by moving the upper and lower walls, and the confining pressure is controlled by moving the left and right walls. Under eccentric loading, five loading walls are built around the specimen. Under uniaxial compression, the two sides and the upper right walls are deleted after the specimen is pre-molding, and the uniaxial compression is realized through the remaining two walls. When biaxial compression is conducted, axial stress is applied through the lower wall and the upper left wall after pre-molding, while the other walls are subject to confining pressure. The schematic diagram of eccentric load uniaxial compression and biaxial compression loading is shown in Fig. 3.

### Determination of meso parameters

**Figure 4:**
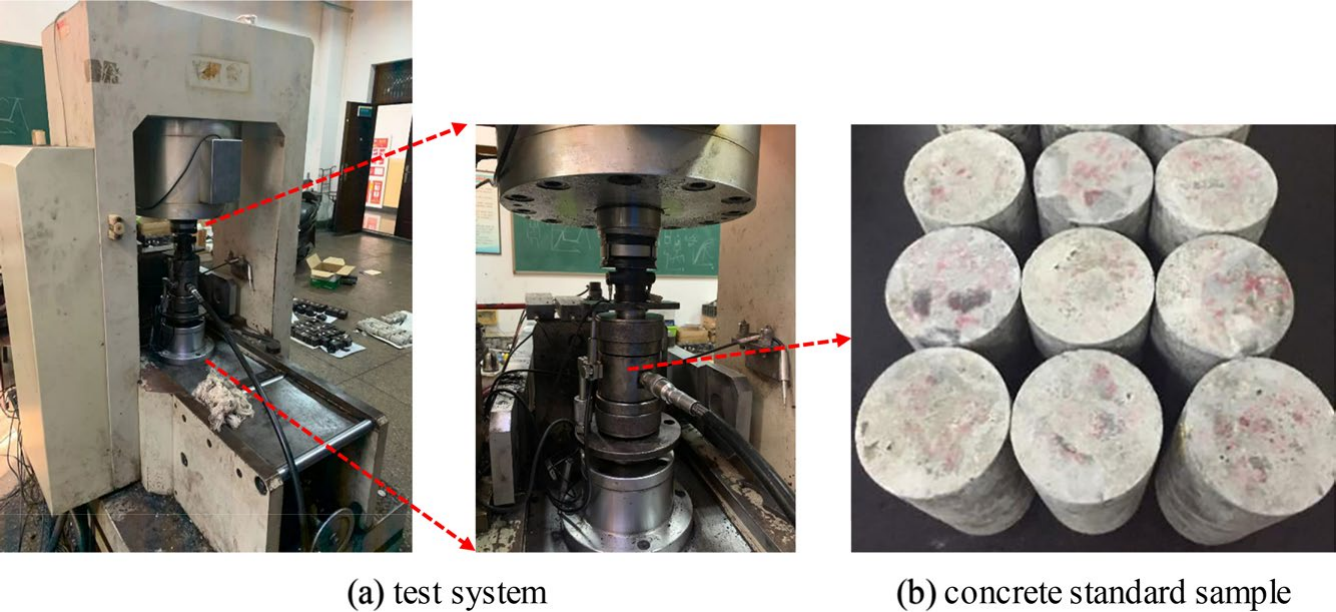
Test equipment and concrete samples.

**Figure 5:**
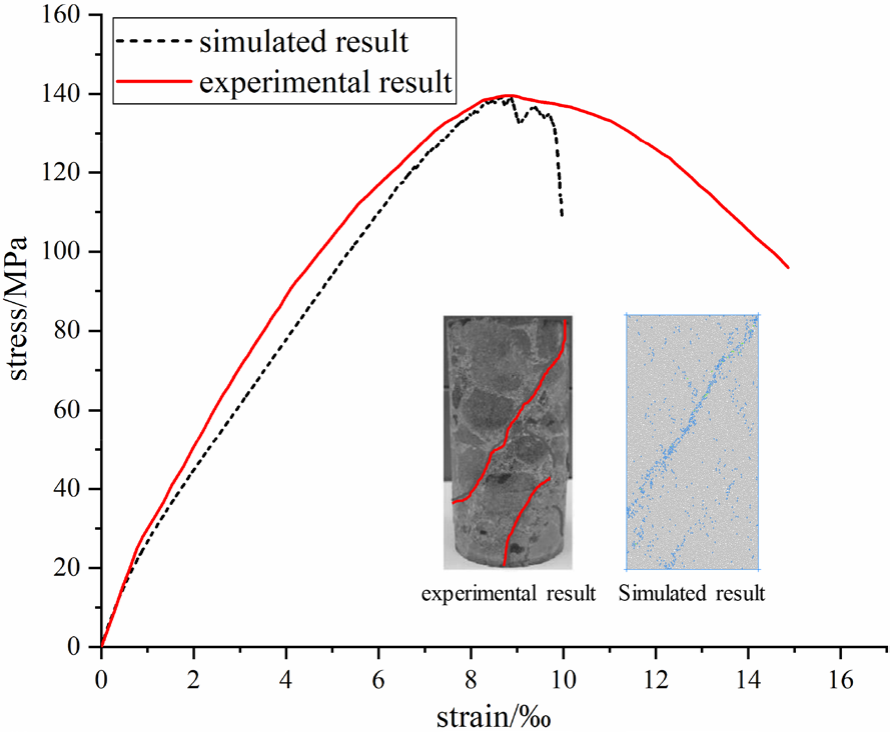
The stress–strain curve of numerical simulation and test.

In the parallel bonding model, several mesoscopic parameters are involved, such as normal stiffness, tangential stiffness, friction coefficient and so on. These parameters will not only change the deformation and strength characteristics of the simulated object, but also affect its final failure mode, so it is necessary to calibrate the parameters according to the laboratory test results^18,19^. High-strength concrete samples with strength grade C60 were used in the laboratory test. The mix proportion for C60 concrete is shown in Table 1. After curing the concrete for 28 days under standard conditions, the 50 mm × 100 mm cylindrical sample was prepared by drilling, cutting and grinding (Fig. 4b). The test equipment used is RMT-150B electro-hydraulic servo testing machine (Fig. 4a), which can apply the maximum axial pressure of 1000kN and the maximum confining pressure of 50 MPa. The testing machine is a special equipment for rock, concrete mechanical properties parameters test research, with uniaxial compression, triaxial compression, direct tensile, direct shear and other functions, test data automatic acquisition, real-time display, after the end of the test can output load deformation curve. In the test process, the sample is preloaded by displacement controlled loading method (the stress level is 0.5 MPa) at first, and then the confining pressure is applied to 20 MPa. When the confining pressure reached a predetermined value, the pressure was stabilized, and the stabilizing time was 1 min. Finally, axial compression was applied. In order to obtain a relatively stable stress–strain curve of concrete, displacement control was adopted in the process of axial compression with a speed of 0.01 mm/min. Figure 5 shows the stress–strain curve and failure mode of concrete obtained from laboratory tests.

**Table 1:** The mix proportion for C60 concrete.

Cement/kg	Additives/kg	Cementing agent/kg	Quartz sand/kg	Coarse aggregate/kg	Water/kg	Quartz sand rate
394	157	551	603	1027	145	37%

The numerical simulation test of concrete under the same conditions as the laboratory test is carried out, and the simulation results are compared with those of the laboratory test. By continuously adjusting the meso parameters in the numerical simulation, until the macro mechanical parameters obtained by the numerical simulation are consistent with the indoor test results. The stress–strain curves obtained by numerical simulation and laboratory test and the failure mode of the sample are shown in Fig. 5. The stress–strain curves obtained by numerical simulation and laboratory test are basically consistent, and the slope of the curve in elastic stage and the peak stress of the sample are approximately equal. The distribution of failure cracks in the sample is basically consistent. The meso parameters of successful calibration are shown in Table 2.

**Table 2:** The micro-parameters used in the PFC 2D model for concrete.

Micro-parameters	Values
particle diameter ratio, $R_{rat} = R_{\max } /R_{\min }$	1.64
Particle friction coefficient, $\mu$	0.3
Density, $\rho$ (kg/m^3^)	1700
Ratio of normal to shear stiffness of the particle, $k_{n} /k_{s}$	2.95
Young’s modulus of the particle, $E_{c}$ (GPa)	60
Ratio of normal to shear stiffness of the parallel bond, $k_{n} /k_{s}$	2.95
Young’s modulus of the parallel bond, $E_{c}$ (GPa)	60
Parallel bond cohesion, mean, $pb_{ - } coh$ (GPa)	1.2
Parallel bond cohesion, standard deviation (MPa)	50
Parallel bond tensile strength, $pb_{ - } ten$ (GPa)	1.0
Parallel bond tensile strength, standard deviation (MPa)	50

## Test results and discussion

### Influence of confining pressure on mechanical parameters

**Figure 6:**
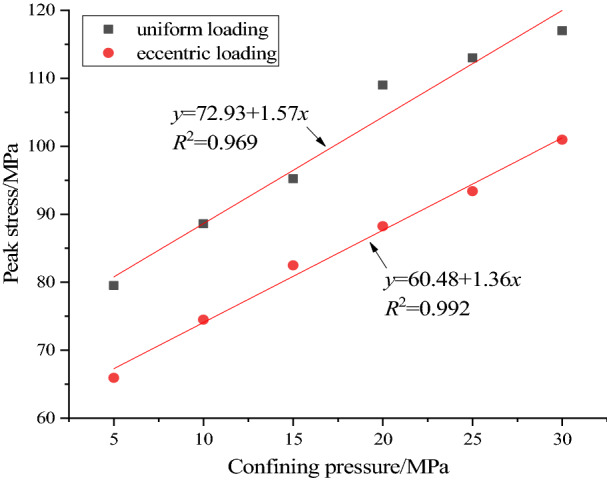
Effect of confining pressure on peak stress.

Figure 6 shows the evolution of the peak stress of concrete with the increase of confining pressure. It can be seen from Fig. 6 that the peak stress of concrete under the action of uniform load and eccentric load increases gradually with the increase of confining pressure, and both show the law of linear function. The slope of fitting curve between peak stress and confining pressure under uniform load is higher than that under eccentric load, which indicates that the peak strength of concrete under uniform load is more sensitive to confining pressure. Under the same confining pressure, the peak stress of concrete under uniform load is higher than that under eccentric load, which indicates that eccentric load is more likely to lead to concrete failure. This is because when the eccentric load is biaxially compressed, the sample can be divided into two parts in the axial direction, the loading area and the non-loading area (Fig. 3). Before the load is applied, the two parts first reach the same confining pressure value, then the load in the loading area gradually increases until the specimen is broken, and the non-loading area maintains a constant confining pressure. In the process of increasing the load in the loading area, there is a stress difference between the loading area and the non loading area, and there is an obvious stress concentration zone near the interface between the two parts. The existence of the stress concentration zone makes the specimen more prone to failure under biaxial compression under eccentric load. The existence of confining pressure can only increase the ability of specimen to resist failure, but it will not eliminate the effect of stress concentration zone under eccentric load, so the peak stress under eccentric load is less than that under uniform load under different confining pressure load.

**Figure 7:**
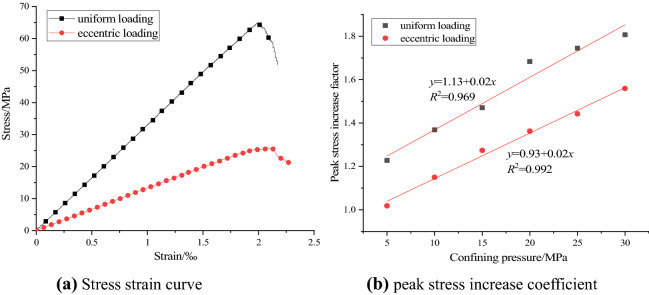
Simulation results of uniaxial compression and stress increase coefficient.

Before carrying out the numerical tests of concrete under different confining pressures, the deformation and failure processes of concrete under uniaxial compression of uniformly distributed load and eccentric load are simulated by means of particle flow simulation software. Figure 7a shows the stress–strain curves of the two loading modes. It can be seen from Fig. 7a that the peak stress of uniaxial compression concrete under uniform load is 64.75 MPa, and the peak stress of uniaxial compression concrete under eccentric load is 25.79 MPa, the former is about 2.5 times of the latter. It can be seen that the existence of eccentric load will seriously reduce the compressive capacity of concrete, and concrete engineering is more prone to safety accidents. In the yield stage, the number and degree of fluctuation of the stress curve under eccentric load are greater than those under uniform load, and there is obvious strain softening phenomenon near the peak time. Similar to biaxial compression, uniaxial compression under eccentric load can be divided into loading area and non loading area. With the increase of axial load in the loading area, there will be obvious stress concentration between the interface of the two areas. The existence of stress concentration promotes the failure of the sample, resulting in the peak stress of the sample under eccentric load is less than the uniform load.

The ratio of peak stress to uniform load in biaxial compression is defined as the peak stress increase coefficient. Under biaxial compression with uniformly distributed load, the increase factor of peak stress increases from 1.2278 at confining pressure 5 MPa to 1.80695 at 30 MPa, which increased by about 1.47 times. The peak stress increase coefficient of eccentric load under biaxial compression increased from 1.018 at confining pressure 5 MPa to 1.559 at 30 MPa, which increased by about 1.53 times. Figure 7b shows the relationship between the peak stress increase factor and the confining pressure under the two loading modes. It can be seen from Fig. 7b that with the increase of confining pressure, the increase coefficients of peak stress under uniform load and eccentric load increase gradually, and they all show the law of linear function. The slope of the two fitting curves is basically the same, which indicates that the variation of the peak stress increase coefficient under the two loading modes is approximately the same, but under the same confining pressure, the peak stress increase coefficient under uniform load compression is higher than that under eccentric load compression.

**Figure 8:**
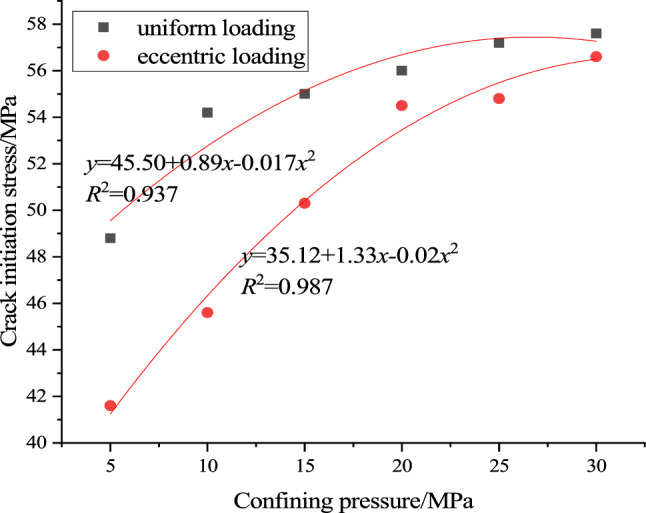
Effect of confining pressure on crack initiation stress.

In this paper, according to the method recommended by potyondy and cundall, the crack initiation stress of concrete is determined according to the change of the number of cracks in the simulation process. The number of cracks *n* in the sample at the peak stress is determined first, and then the stress corresponding to 1% of *n* is found as the crack initiation stress of the sample. According to the above method, the crack initiation stress of concrete under uniform load and eccentric load compression under different confining pressure conditions is determined, as shown in Fig. 8.

It can be seen from Fig. 8 that the crack initiation stress of concrete under uniform load and eccentric load compression increases gradually with the increase of confining pressure, and the relationship is quadratic function. Under the same confining pressure, the crack initiation stress of concrete under eccentric load is less than that under uniform load, which indicates that the crack initiation evolution of concrete under eccentric load is more likely to occur, resulting in the failure of samples. This is of great significance for the determination of monitoring and early warning indexes of concrete engineering under eccentric load. Under eccentric loading, the stress concentration band between the loaded and unloaded regions makes the specimen easier to form meso cracks in this region. The end effect of the loading plate and the existence of initial damage will amplify the stress concentration phenomenon in this area. Meso-cracks first appear in these two parts (Fig. 15), which causes the sample cracking stress to be less than the uniform load when the eccentric load is applied.

### Influence of confining pressure on failure mode

**Figure 9:**
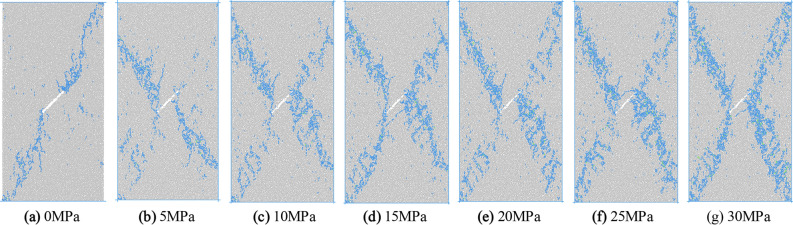
Failure mode of concrete under uniform load.

**Figure 10:**
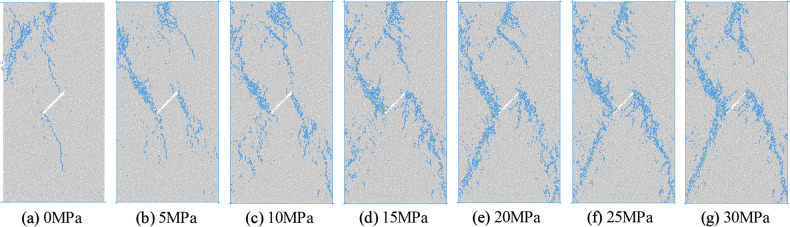
Failure mode of concrete under eccentric load.

In order to analyze the influence of loading mode and confining pressure on the failure mode of concrete, the failure mode diagram at the time when the stress of the sample is 80% of the peak stress after failure is extracted, as shown in Figs. 9 and 10.

It can be seen from Figs. 9 and 10 that the failure mode of concrete is affected by the initial damage, loading mode and confining pressure at the same time. Under uniaxial compression with uniformly distributed load, the macro failure crack direction of the sample is approximately parallel to the initial crack. The macro crack is connected with both ends of the initial crack and runs through the upper and lower ends of the sample. The tensile shear composite failure of the sample occurs, and the simulation results are consistent with the research results of Zhang^20^. When the confining pressure is 5 MPa, the macro cracks of the sample are approximately vertical to the initial cracks, and they are in the state of anti-wing cracks. The macro cracks do not reach the end of the sample, but penetrate from both sides of the sample, and the shear failure of the sample occurs. When the confining pressure is higher than 10 MPa, the shear failure of the specimen occurs mainly, and the macro cracks are X-shaped, and the turning points of the cracks on the left and right sides are located at the two tips of the initial cracks. With the increase of confining pressure, the distribution range and width of the macro cracks increase.

When the specimen is subjected to uniaxial compression under eccentric load, the macro cracks are located in the direct loading area. There is a mode I shear crack approximately parallel to the loading direction in the middle of the specimen, which is connected with both ends of the initial crack and does not penetrate the specimen. Finally, the failure of the specimen is caused by the crack starting from the interface between the loading area and the non-loading area, and the inclined crack cuts out from the middle of the sample. After confining pressure is applied, due to the limitation of confining pressure, it is difficult for the crack initiated from the stress concentration position at the junction of loading area and non-loading area to propagate to the edge of the specimen, which changes into the increase of the failure range in this area. Due to the joint influence of confining pressure and loading area, some cracks are also produced in the non-loading zone. The macro cracks formed in the non-loading zone are inclined cracks connecting the end of the initial crack and the end of the specimen, while the through cracks leading to the failure of the specimen are still located in the loading zone. The through cracks are broken line shear cracks with the initial crack as the turning point.

The macro cracks formed in the non-loading zone are inclined cracks connecting the initial crack ends and the end of the sample, and the cracks that cause the specimen damage are still located in the loading zone. The through cracks are the broken line shear cracks with the initial cracks as the turning point.

### Effect of confining pressure on the evolution of microcracks

**Figure 11:**
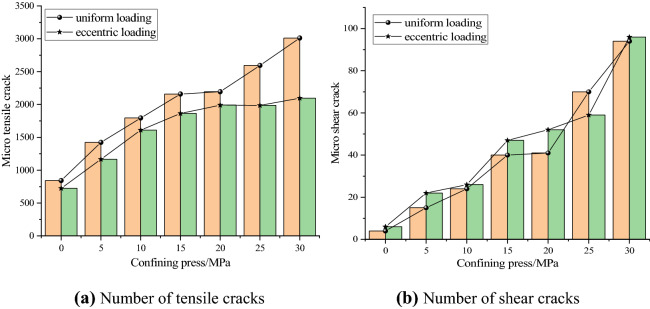
Relationship between crack number and confining pressure.

In order to compare and analyze the difference of the number of microcracks under uniform load and eccentric load, the number of microcracks in the sample was counted when the post peak stress dropped to 80% of the peak stress. Figure 11 shows the relationship between the number of tensile cracks, shear cracks and confining pressure of concrete under uniform and eccentric loads. It can be seen from Fig. 11 that with the increase of confining pressure, the number of tensile cracks and shear cracks under the two loading modes increases gradually, and both are higher than that under uniaxial compression. Under the same confining pressure, the number of tensile cracks under uniform load is higher than that under eccentric load, and with the increase of confining pressure, the increase of the number of tensile cracks under the two loading methods can be divided into two stages: deceleration growth and acceleration growth. Except for the confining pressure of 25 MPa, the number of shear cracks caused by eccentric load is higher than that of uniform load. By adding the number of tensile cracks and shear cracks, the number of cracks under uniform load is higher than that under eccentric load. The relationship between the total number of cracks shows that there is a stress concentration area in the specimen under eccentric load. The guiding effect of stress concentration leads to the rapid formation and propagation of microcracks, which leads to the failure of specimens. That is to say, concrete is more prone to failure under eccentric load. Therefore, the eccentric load should be avoided as far as possible in the construction process; otherwise, the influence of eccentric load should be considered in the support design of the tunnel or roadway project under eccentric load, and the stress concentration area caused by eccentric load should be mainly supported.

**Figure 12:**
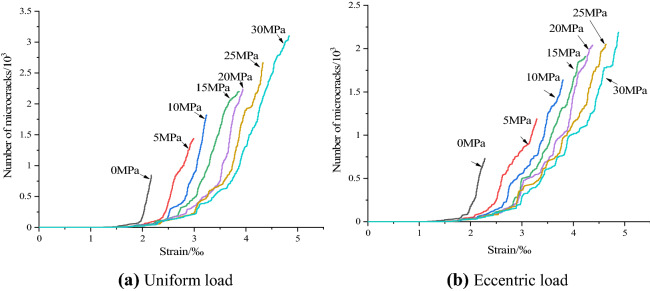
Relationship between crack number and axial strain.

Figure 12 shows the evolution trend of crack number with axial strain under different confining pressures under uniform and eccentric loads. It can be seen from Fig. 12 that with the increase of confining pressure, the strain value corresponding to the inflection point of the curve increases gradually, and the number of cracks also increases gradually. The difference is that the increase of axial strain under uniform load and different confining pressure is more balanced, while under eccentric load, the increase of axial strain gradually decreases with the increase of confining pressure, and the increase of the number of microcracks also decreases.

**Figure 13:**
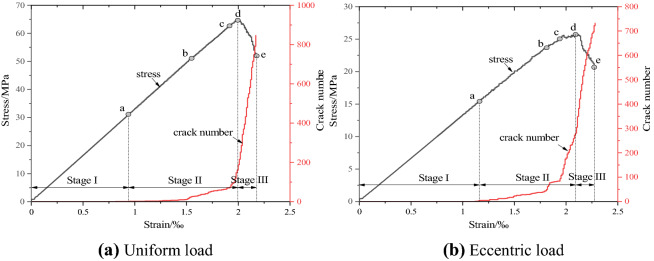
Relationship between crack number and stress–strain evolution under uniaxial compression.

It can be seen from Fig. 12 that, under different confining pressures, the variation law of the relationship curve between the number of meso-cracks and the axial strain of concrete samples under uniform load and eccentric load is basically the same, which only shows the difference of the total number of cracks. Considering the limitation of the length of the paper, we only take the evolution curve of the number of cracks at 20 MPa as an example to analyze the micro-evolution process of cracks. In order to systematically compare the damage process of concrete under the two loading modes, this paper presents the fracture process of concrete with initial damage under uniaxial compression and confining pressure of 20 MPa under uniform load and eccentric load. Figure 13 shows the evolutionary process of the number of cracks with the stress–strain curve during uniaxial compression under uniform and eccentric loads. According to the evolution law of stress–strain and the number of microcracks in Fig. 13, the propagation of microcracks in concrete during uniaxial compression is divided into three stages: the calm period I at the initial loading stage, the pre-peak expansion period II from the crack initiation point to the peak point, and the rapid increase period III after the peak.

**Figure 14:**
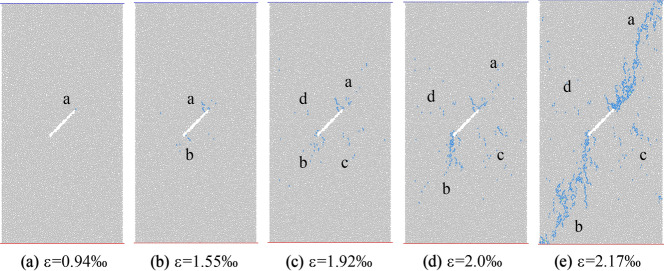
Failure process of specimen under uniaxial compression under uniform load.

**Figure 15:**
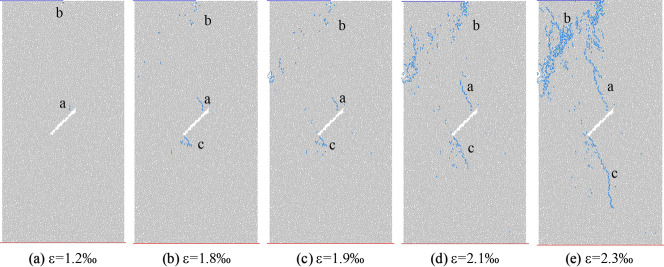
Failure process of specimen under uniaxial compression under eccentric load.

According to the turning point of the stress–strain region and the number of microcracks evolution curve, five times a–e are selected in Fig. 13 to observe the propagation and evolution process of microcracks, where time a corresponds to the crack initiation stress position. Figures 14 and 15 show the crack growth process of uniform load and eccentric load at different loading time.

It can be seen from Fig. 14 that the microcrack starts from the upper end of the initial crack due to the stress concentration at the tip of the initial crack when loading to moment a. As the stress continues to increase, to the moment b, crack a further expands, and a small number of associated cracks appear. Crack b and its associated cracks appear at the lower tip of the initial crack. When loading to moment c, the formed a and b cracks continue to extend to the end of the specimen, the associated cracks increase further, and a small number of c and d cracks appear on both sides of the initial crack. At the time of loading d, the crack a does not change obviously, the crack b approaches the end of the specimen after propagation, and the number of cracks c and d increases slightly. At the time e after the peak, both cracks a and b extend to the end of the specimen, and the final cracks a and b are approximately parallel to the initial crack direction.

It can be seen from Fig. 15 that at the crack initiation stress point a, due to the stress concentration at the end of the upper loading plate and the end of the initial crack, micro cracks a and b appear in both regions. Different from the uniform load, the crack a does not appear at the tip of the initial crack, but appears on the right side of the initial crack tip, which is in the same vertical position as the crack b at the end of the loading plate. At the moment b, cracks a and b propagate gradually, and crack c appears at the tip of the initial crack. At the moment c, the change of crack a and crack c is not obvious, and crack b is expanding continuously, and the crack formed on the left side of the sample is in the trend of penetration. At the peak point d, the crack b runs through the left side of the specimen, the cracks a and c further expand and elongate, and the scattered meso cracks appear on the left side of the crack c. At the time e after the peak, the width of crack b increases further, cracks a and c extend further, crack a runs through crack b, and the propagation directions of cracks a and c are approximately parallel to the axial direction.

**Figure 16:**
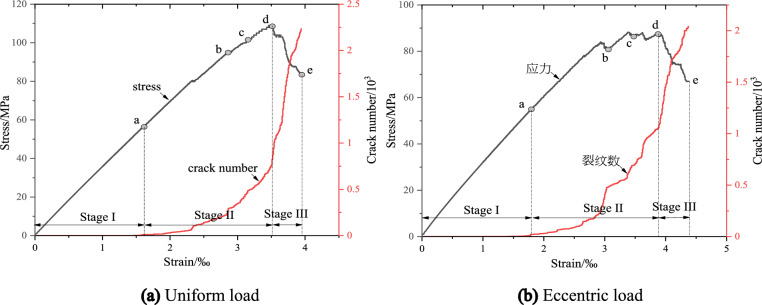
Relationship between crack number and stress–strain evolution under triaxial compression.

Figure 16 shows the evolution of crack number with stress–strain curves during biaxial compression (confining pressure 20 MPa) under uniform load and eccentric load. According to the evolution law of stress–strain and number of microcracks in Fig. 16, the propagation process of microcracks in concrete under biaxial compression can be divided into three stages, quiescence stage I at the initial stage of loading, pre-peak propagation stage II from crack initiation point to peak point, and post peak rapid growth stage III.

**Figure 17:**
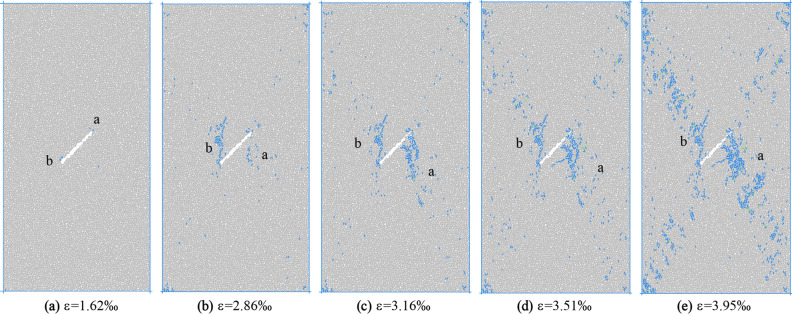
Crack evolution under biaxial compression under uniform load.

**Figure 18:**
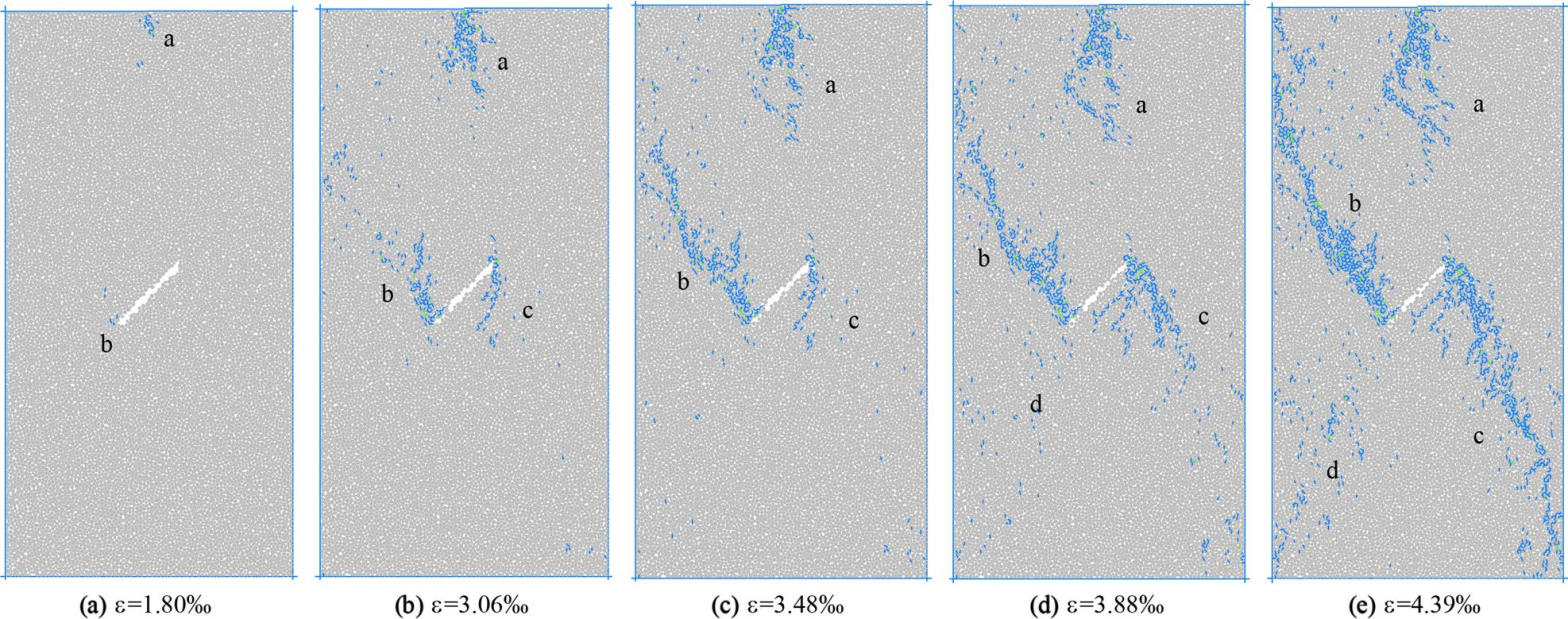
Crack evolution under biaxial compression under eccentric load.

According to the turning point of the stress–strain region and the number of microcracks evolution curve, five times a–e are selected in Fig. 16 to observe the propagation and evolution process of microcracks, where time a corresponds to the crack initiation stress position. Figures 17 and 18 show the crack growth process of uniform load and eccentric load at different loading time. It can be seen from Fig. 17 that when the uniformly distributed load is compressed, cracks a and b appear at both ends of the initial crack when the load reaches time a. At the moment b, the crack a and b propagate parallel to the axial direction of the loading, in which the crack b at the lower tip of the initial crack propagates upward, and the crack a at the upper tip propagates downward in the shape of an anti-wing crack. When the loading continues to c, the length of crack b changes greatly, the crack continues to extend and the width increases, and there are scattered micro cracks in the diagonal position of the sample. At peak d, the number of cracks in the diagonal position of the sample increases further, showing a trend of penetration. At the moment e after peak, the cracks at the diagonal of the sample are connected with cracks a and b, and the crack width increases with the increase of stress. It can be seen from Fig. 18 that the crack initiation and propagation process under eccentric load is different from that under uniform load. At moment a, the crack starts and propagates from the end of loading plate and the lower tip of initial crack. At the moment of loading to b, crack a continues to expand downward and its width increases with the increase of load, crack b expands upward and its width increases, and crack c forms at the top of the initial crack and propagates downward. At the moment c, the length of crack a and b increases slightly, mainly in the form of width, and the crack c changes little. At the time of peak d, crack a does not change much, crack b extends to the right edge of the sample, scattered microcracks appear in the d area of the lower left corner of the sample, and crack c continues to expand to the lower right corner. At the time e after the peak, cracks b and c together with the initial cracks form a through crack, and the sample is destroyed.

## Conclusion

1. Under the condition of uniform load and eccentric load, the peak stress of concrete increases linearly with the increase of confining pressure. The crack initiation stress increases as a quadratic function with the increase of confining pressure. At the same confining pressure, the peak stress and crack initiation stress of specimen under uniform load compression are higher than those under eccentric load compression.
2. The peak stress increase factor is defined, and under the same confining pressure, the peak stress increase coefficient of uniform load compression is higher than that of eccentric load compression. With the increase of confining pressure, the peak stress increase coefficients of the two loading modes increase linearly, and the increase rates are approximately equal.
3. The failure mode of concrete is influenced by initial damage, loading mode and confining pressure. Under uniaxial compression with uniformly distributed load, the macro fracture surface consists of the initial crack and the crack in the same direction as the initial crack. Under uniaxial compression with eccentric load, the cracks are located at one side of the loading zone, and mode I shear cracks are formed which are approximately parallel to the loading direction.
4. With the increase of confining pressure, the failure mode of concrete under uniform load and eccentric load has little change. Under the condition of uniform load, the concrete mainly occurs X-type shear failure, and the X-type turning point is the two tips of the initial crack. Under the condition of eccentric load, the concrete mainly occurs broken line shear failure, and the main cracks are located in the loading area. Under the influence of the loading area and confining pressure, an inclined crack will also appear in the non-loading area.
5. The evolution process of the number of microcracks in concrete under the conditions of uniform load and eccentric load can be divided into three stages: the calm period I at the initial loading stage, the pre-peak expansion period II from the crack initiation point to the peak point, and the rapid increase period III after the peak, and the evolution process of microcrack propagation in different stages is analyzed in detail.

## Data Availability

The datasets used or analyzed during the current study are available from the corresponding author on reasonable request.

## References

[1] Wang XH (2018). Initial damage effect on dynamic compressive behaviors of roller compacted concrete (RCC) under impact loadings. Constr. Build. Mater..

[2] Zhang YF, Xia XZ, Wu ZJ, Zhang Q (2020). The effect of initial defects on overall mechanical properties of concrete material. CMC-Comput. Mat. Contin..

[3] Yoon JS, Zang A, Stephansson O (2012). Simulating fracture and friction of Aue granite under confined asymmetric compressive test using clumped particle model. Int. J. Rock Mech. Min. Sci..

[4] Wang T, Zhao H, Ge L, Zhang H, Liu R (2020). Study on deformation evolution law of cox under asymmetric loading by digital image correlation. Appl. Optics.

[5] Yu Z, Huang Q, Shan Y, Ren Z (2018). Failure criterion of ordinary concrete subjected to triaxial compression of full section and local loadings. J. Mater. Civ. Eng..

[6] Zingg L, Briffaut M, Baroth J, Malecot Y (2016). Influence of cement matrix porosity on the triaxial behaviour of concrete. Cem. Concr. Res..

[7] Wang H, Wang L, Song Y, Wang J (2016). Influence of free water on dynamic behavior of dam concrete under biaxial compression. Constr. Build. Mater..

[8] Tian W, Han N (2019). Analysis on meso-damage processes in concrete by X-ray computed tomographic scanning techniques based on divisional zones. Measurement.

[9] Ansari A, Mahajan MM (2020). Performance based simulation of pervious concrete using discrete element method. Gradev..

[10] Lian CQ, Yan ZG, Beecham S (2011). Numerical simulation of the mechanical behaviour of porous concrete. Eng. Comput..

[11] Huang YH, Yang SQ, Ranjith PG, Zhao J (2017). Strength failure behavior and crack evolution mechanism of granite containing pre-existing non-coplanar holes: Experimental study and particle flow modeling. Comput. Geotech..

[12] Song ZY, Konietzky H, Herbst M (2019). Three-dimensional particle model based numerical simulation on multi-level compressive cyclic loading of concrete. Constr. Build. Mater..

[13] Zhang YY, Shao ZS, Wei W, Qiao RJ (2019). PFC simulation of crack evolution and energy conversion during basalt failure process. J. Geophys. Eng..

[14] Haeri H, Sarfarazi V, Marji MF (2020). Numerical simulation of the effect of confining pressure and tunnel depth on the vertical settlement using particle flow code (with direct tensile strength calibration in PFC Modeling). Smart. Struct. Syst..

[15] Yao W (2019). Experimental and numerical study on mechanical and cracking behaviors of flawed granite under triaxial compression. Measurement.

[16] Zhang XP, Wong LNY (2013). Loading rate effects on cracking behavior of flaw-contained specimens under uniaxial compression. Int. J. Fract..

[17] Yang SQ, Huang YH, Ranjith PG, Jiao YY, Ji J (2015). Discrete element modeling on the crack evolution behavior of brittle sandstone containing three fissures under uniaxial compression. Acta Mech. Sin..

[18] Ajamzadeh MR, Sarfarazi V, Haeri H, Dehghani H (2018). The effect of micro parameters of PFC software on the model calibration. Smart. Struct. Syst..

[19] Wang WC (2019). Experimental and numerical study on failure modes and shear strength parameters of rock-like specimens containing two infilled flaws. Int. J. Civ. Eng..

[20] Zhang XP, Wong LNY (2012). Cracking processes in rock-like material containing a single flaw under uniaxial compression: A numerical study based on parallel bonded-particle model approach. Rock Mech. Rock Eng..

